# Genetic predisposition for increased red blood cell distribution width is an early risk factor for cardiovascular and renal comorbidities

**DOI:** 10.1242/dmm.044081

**Published:** 2020-05-18

**Authors:** Xi Cheng, Blair Mell, Ahmad Alimadadi, Sarah Galla, Cameron G. McCarthy, Saroj Chakraborty, Venkatesha Basrur, Bina Joe

**Affiliations:** 1 Center for Hypertension and Precision Medicine, Program in Physiological Genomics, Department of Physiology and Pharmacology, University of Toledo College of Medicine and Life Sciences, Toledo, OH 43614, USA; 2 Department of Pathology, University of Michigan, Ann Arbor, MI 48109, USA

**Keywords:** Red blood cell distribution width, Cardiovascular, Renal, Hematologic, Genetics, Proteomics

## Abstract

Red blood cell distribution width (RDW) is a measurement of the variation in size and volume of red blood cells (RBCs). Increased RDW, indicating a high heterogeneity of RBCs, is prominently associated with a variety of illnesses, especially cardiovascular diseases. However, the significance of this association to the onset and progression of cardiovascular and renal diseases is unknown. We hypothesized that a genetic predisposition for increased RDW is an early risk factor for cardiovascular and renal comorbidities. Since there is no known animal model of increased RDW, we examined a CRISPR/Cas9 gene-edited rat model (Rffl^TD^) that presented with features of hematologic abnormalities as well as severe cardiac and renal comorbidities. A mass spectrometry-based quantitative proteomic analysis indicated anemia of these rats, which presented with significant downregulation of hemoglobin and haptoglobin. Decreased hemoglobin and increased RDW were further observed in Rffl^TD^ through complete blood count. Next, a systematic temporal assessment detected an early increased RDW in Rffl^TD^, which was prior to the development of other comorbidities. The primary mutation of Rffl^TD^ is a 50 bp deletion in a non-coding region, and our study has serendipitously identified this locus as a novel quantitative trait locus (QTL) for RDW. To our knowledge, our study is the first to experimentally pinpoint a QTL for RDW and provides a novel genetic rat model mimicking the clinical association of increased RDW with poor cardio-renal outcome.

## Abstract (precis)

**Summary:** Our study pinpoints a quantitative trait locus for red blood cell distribution width (RDW) and provides a novel genetic rat model mimicking the clinical association of increased RDW with poor cardio-renal outcome.

## INTRODUCTION

Red blood cell distribution width (RDW) is a measure of the range of variation in size and volume of red blood cells (RBCs). Increased RDW, as reported by the standard complete blood count (CBC), represents an increased heterogeneity of RBCs and it is also known as anisocytosis, referring to a patient's RBCs of unequal size. Increased RDW is noted as a feature associated with a variety of diseases, including cancer (Koma et al., 2013) and metabolic syndrome (Sanchez-Chaparro et al., 2010). Increased RDW has been also observed to co-exist with multiple cardiovascular disorders, such as hypertension (Bilal et al., 2016), atrial fibrillation (Shao et al., 2018) and heart failure (Lippi et al., 2018). However, little is known regarding the significance of this association. Lack of animal models with a prominent RDW phenotype precludes studies from examining the relationship between RDW and the co-morbid cardiovascular conditions.

We previously reported a CRISPR/Cas9-based genome-engineering rat model with a genomic deletion occurring within a long non-coding RNA, *Rffl-lnc1*, which is located within the 5′UTR intronic region of the rififylin (*Rffl*) gene (Cheng et al., 2017). This targeted disruption model, referred to as Rffl^TD^, generated on the genomic background of the inbred Dahl salt-sensitive rat (S), had a 50 bp deletion within *Rffl-lnc1* and had significantly higher blood pressure compared with the wild-type S rat (Cheng et al., 2017). In the current study, we performed an unbiased quantitative proteomics approach to understand the genetic mechanism causing cardiovascular dysfunction in Rffl^TD^. Interestingly, our study showed that a genetic predisposition for increased RDW is an early risk factor for subsequent cardiovascular and renal comorbidities in Rffl^TD^, which further supports the clinical observation that increased RDW is associated with poor cardio-renal outcome in patients (Lu et al., 2017). To our knowledge, our study is the first to experimentally pinpoint a quantitative trait locus for RDW and provides a novel genetic rat model with an early genetic onset of increased RDW accompanied by eventual cardio-renal comorbidities.

## RESULTS

### Proteomics study indicating hematologic disorders in Rffl^TD^

**Fig. 1.**
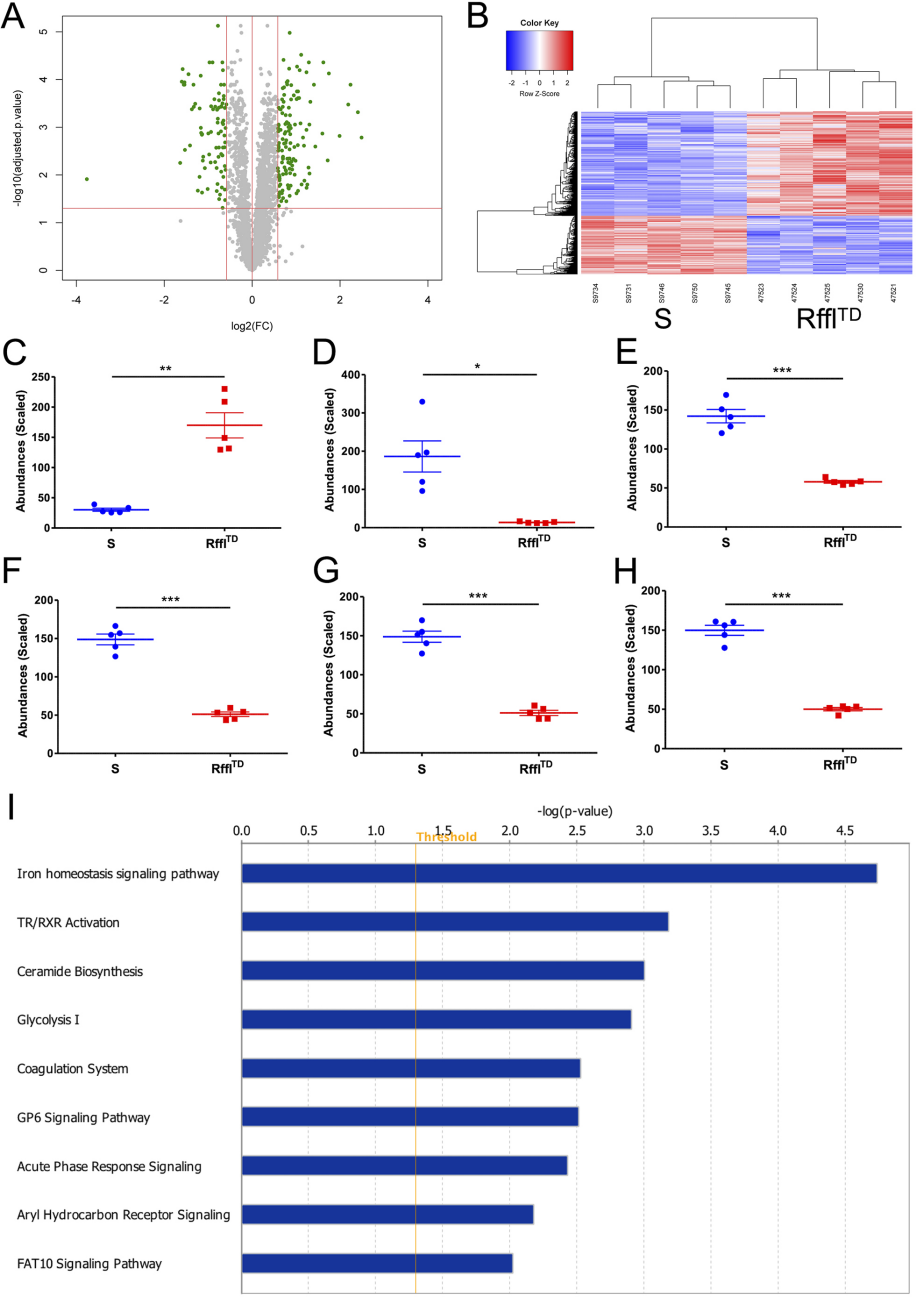
**The proteomic data suggest that Rffl^TD^ is more susceptible than S to heart dysfunction and hematologic disorder.** (A) Volcano plot of proteomic data. The horizontal red line represents a Benjamini–Hochberg adjusted *P*-value with a cut-off of 0.05. All the gray and green circles above this horizontal red line represent the differentially expressed proteins (DEPs) with statistical significance (adjusted *P*<0.05). The vertical red lines mark the limits for fold change with a cut-off value of 1.5, whereby the green circles outside of the area between the two vertical red lines represent the proteins with >1.5-fold change, and the gray circles between the two vertical red lines represent the proteins with <1.5-fold change. (B) Heat map of DEPs between S and Rffl^TD^. (C-H) The quantified abundances of atrial natriuretic peptide (C), haptoglobin (D), hemoglobin subunit alpha 1 (E), hemoglobin subunit alpha 2 (F), hemoglobin subunit beta (G) and hemoglobin subunit epsilon 1 (H). The values in C-H are expressed as mean±s.e.m. *n*=5 rats per group. The Benjamini-Hochberg method was used for the *P*-value adjustment of multiple testing. *P<0.05, **P<0.01, ***P<0.001 (Benjamini–Hochberg adjusted *P*-values from Student's *t*-test). (I) Significant canonical pathways. Only the significant pathways with −log (*P*-value) greater than 2.0 are shown.

An unbiased quantitative proteomics study was performed to identify cardiac proteins that were differentially expressed between the wild-type S rat and the Rffl^TD^ model. Of 3224 protein signals identified by mass spectrometry, 1245 of them were differentially expressed according to the Benjamini–Hochberg method for the *P*-value adjustment of multiple testing (Table S1). The volcano plot of the 3224 proteins and the heat map of the 1245 differentially expressed proteins (DEPs) are shown in Fig. 1A and B, respectively, indicating a clear difference in the protein expression pattern between S and Rffl^TD^. Among all the DEPs, atrial natriuretic peptide, which was reported to be increased in heart failure patients (Brandt et al., 1993), was the top upregulated protein (based on fold change) in Rffl^TD^ (Fig. 1C). Haptoglobin was the top downregulated protein (based on fold change) in Rffl^TD^ (Fig. 1D) and hemoglobin (HGB) subunits, including HGB subunit alpha 1, HGB subunit alpha 2, HGB subunit beta and HGB subunit epsilon 1, were also downregulated in Rffl^TD^ (Fig. 1E-H), suggesting that Rffl^TD^ was prone to an anemic condition. The canonical pathway analysis from Ingenuity Pathway Analysis (IPA) showed that the iron homeostasis signaling pathway was the top significantly dysregulated pathway in Rffl^TD^ (Fig. 1I), further indicating hematologic disorders in Rffl^TD^.

### Hematologic and cardio-renal disorders observed in Rffl^TD^

**Fig. 2.**
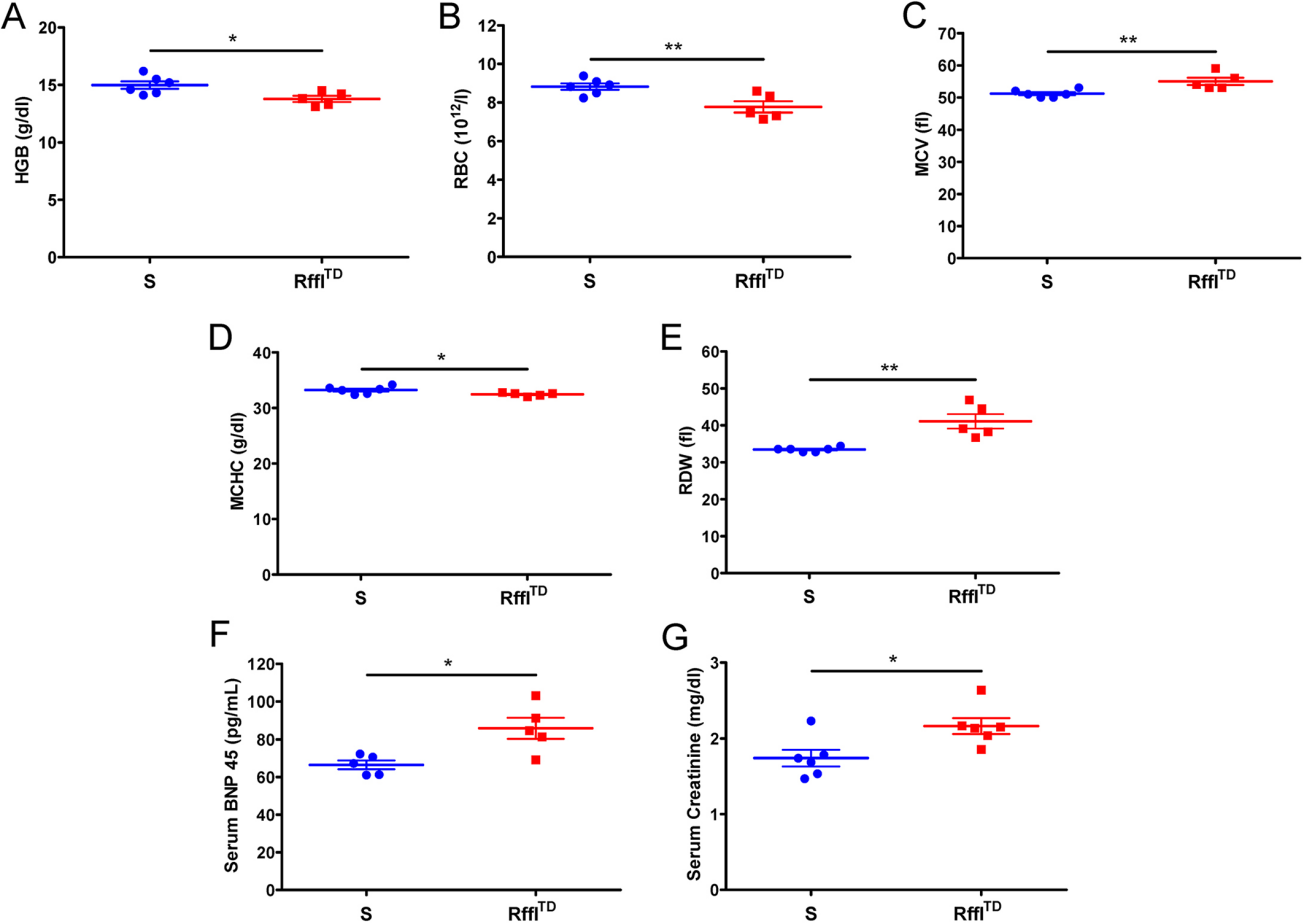
**Hematologic and cardio-renal parameters were significantly worse in Rffl^TD^ compared with S.** (A) Hemoglobin (HGB). (B) Red blood cell (RBC). (C) Mean corpuscular volume (MCV). (D) Mean corpuscular HGB concentration (MCHC). (E) Red blood cell distribution width (RDW). (F) Serum B-type natriuretic peptide (BNP). (G) Serum creatinine. Values are expressed as mean±s.e.m. *P<0.05, **P<0.01 (Student's *t*-test). *n*=5-6 rats (∼15 weeks of age) per group.

To directly assess hematologic condition in Rffl^TD^, a CBC was performed. Compared with S, Rffl^TD^ had lower HGB, fewer RBCs, higher mean corpuscular volume (MCV), lower mean corpuscular HGB concentration (MCHC) and higher RDW (Fig. 2A-E), confirming that hematologic parameters were significantly worse in Rffl^TD^. High levels of serum B-type natriuretic peptide (BNP) and creatinine were further indicative of cardiovascular and renal comorbidities in Rffl^TD^ (Fig. 2F,G).

### Increased RDW occurring prior to the development of other hematologic and cardio-renal disorders in Rffl^TD^

**Fig. 3.**
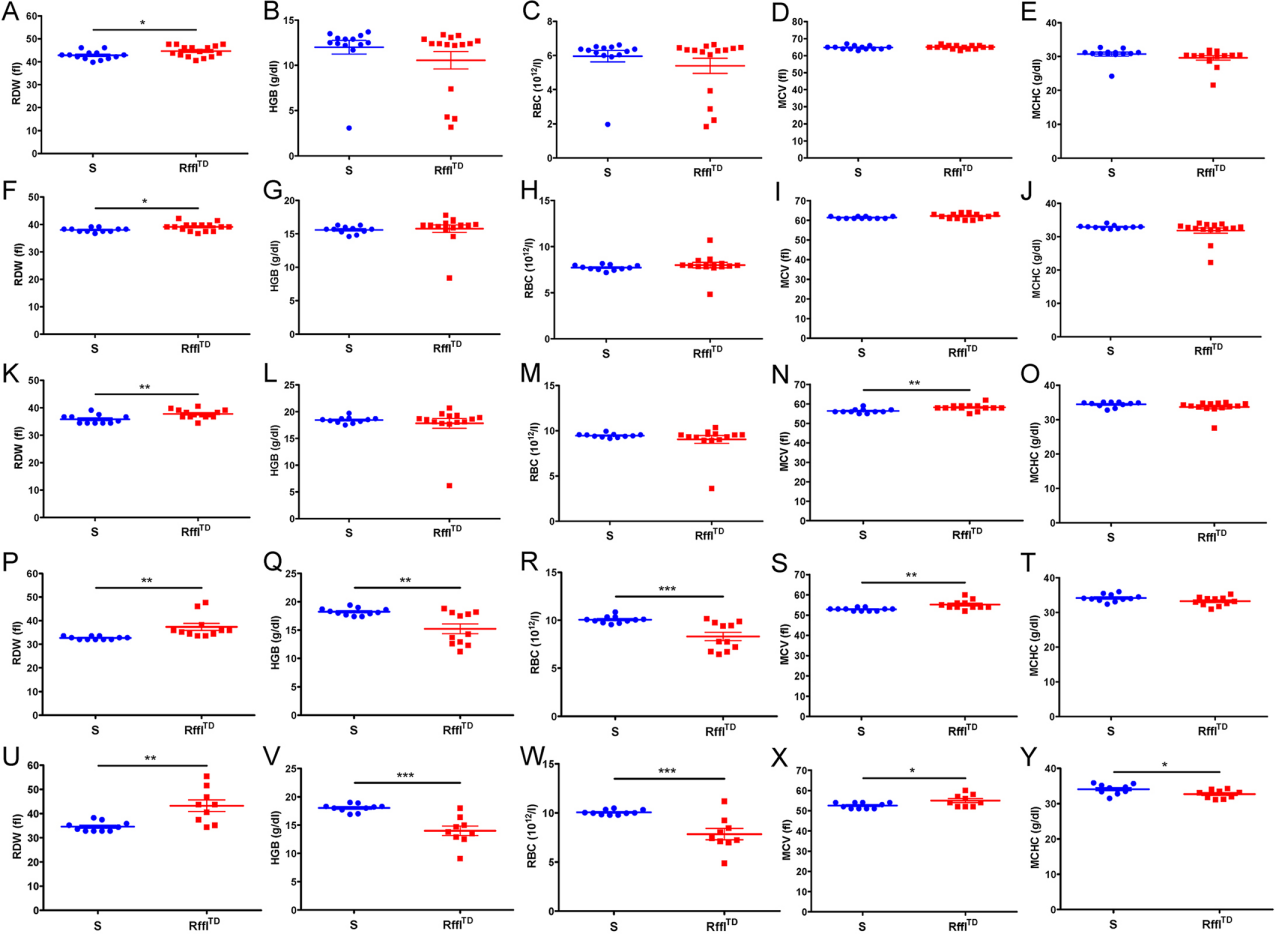
**The progression of hematologic disorder in Rffl^TD^.** (A-Y) RDW, HGB, RBC, MCV and MCHC are shown at various ages: 5 weeks (A-E); 8 weeks (F-J); 11 weeks (K-O); 14 weeks (P-T); 17 weeks (U-Y). Values are expressed as mean±s.e.m. *P<0.05, **P<0.01, ***P<0.001 (Student's *t*-test). *n*=9-15 rats per group.

**Fig. 4.**
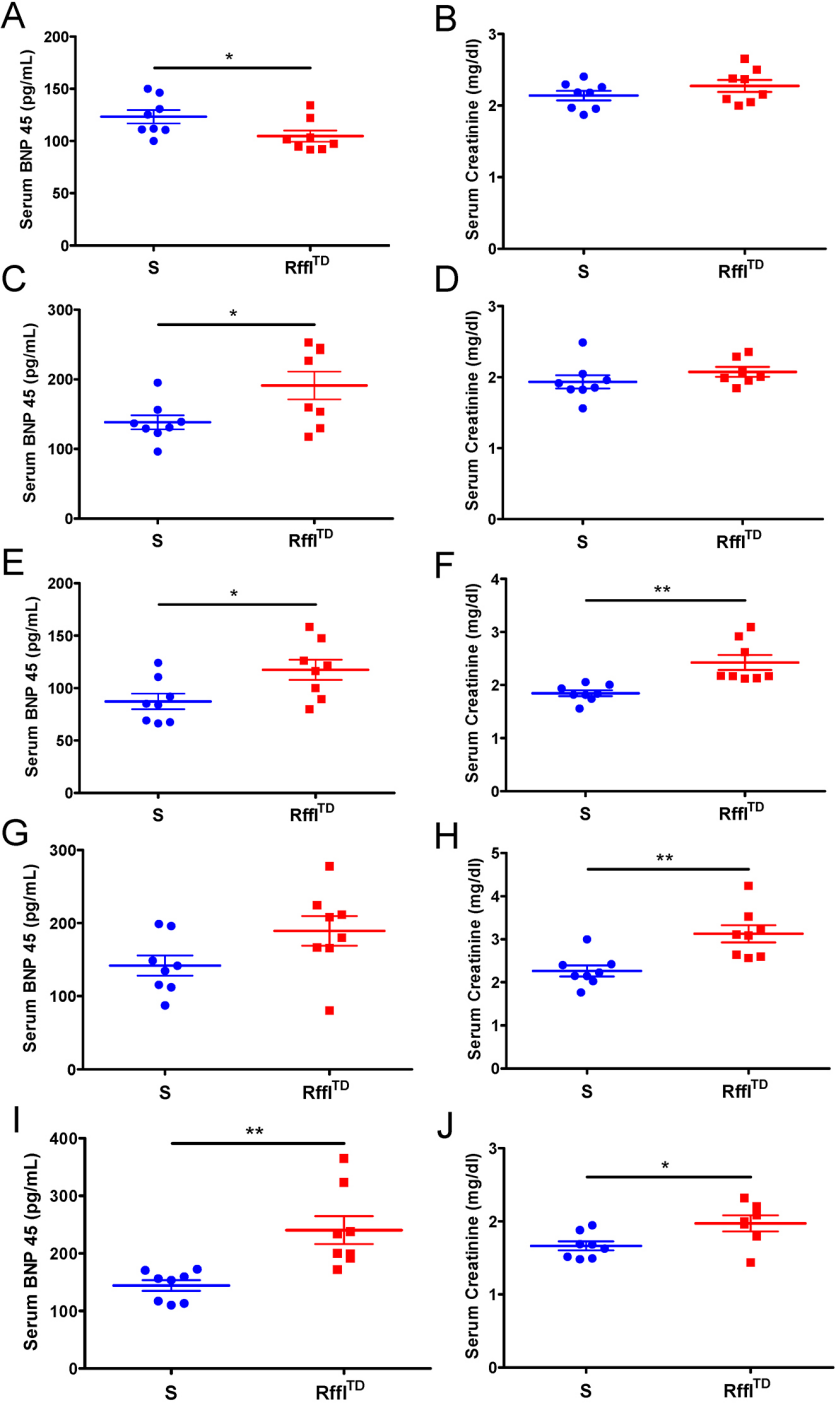
**The progression of cardio-renal dysfunction in Rffl^TD^.** Serum BNP and serum creatinine are shown at various ages: 5 weeks (A,B); 8 weeks (C,D); 11 weeks (E,F); 14 weeks (G,H); 17 weeks (I,J). Values are expressed as mean±s.e.m. *P<0.05, **P<0.01 (Student's *t*-test). *n*=7-8 rats per group.

As hematologic and cardio-renal parameters were both significantly worse in Rffl^TD^ compared with S when they were ∼15 weeks of age (Fig. 2), the temporal cause of the hematologic and cardio-renal disorders was further investigated. Starting at 5 weeks of age, blood was collected every 3 weeks from the same batch of rats for the measurements of CBC, serum BNP and serum creatinine. During the whole temporal study, some rats died naturally or were euthanized due to the discomfort from abnormal bulgy eyes caused by retro-orbital bleeding procedure. At 5 and 8 weeks of age, RDW was significantly higher in Rffl^TD^ and no significant changes were observed in other hematologic parameters (Fig. 3A-J). At 11 weeks of age, RDW and MCV were both significantly higher in Rffl^TD^ than in S, and no significant changes were observed in HGB, RBC and MCHC (Fig. 3K-O). At 14 weeks of age, significant differences in RDW, HGB, RBC and MCV were observed, and only MCHC was not significantly different between Rffl^TD^ and S (Fig. 3P-T). At 17 weeks of age, all the hematologic parameters were significantly different between Rffl^TD^ and S (Fig. 3U-Y), which was consistent with hematologic differences observed in Fig. 2. Interestingly, serum BNP level was significantly higher in S than in Rffl^TD^ at 5 weeks of age (Fig. 4A), indicating that Rffl^TD^ did not show clear cardiac dysfunction at this time point. Starting from 8 weeks of age, serum BNP was significantly higher in Rffl^TD^ than in S (Fig. 4C,E,G,I), indicating that the clear cardiac dysfunction occurred at 8 weeks of age. Serum creatinine level was significantly higher in Rffl^TD^ than in S starting at 11 weeks of age (Fig. 4B,D,F,H,J). Overall, the hematologic and cardio-renal analyses showed that Rffl^TD^ had higher hematologic RDW at 5 weeks of age, followed by cardiac dysfunction starting at 8 weeks of age and renal dysfunction starting at 11 weeks of age, suggesting that increased RDW is an early indicator of cardio-renal dysfunction in Rffl^TD^.

**Fig. 5.**
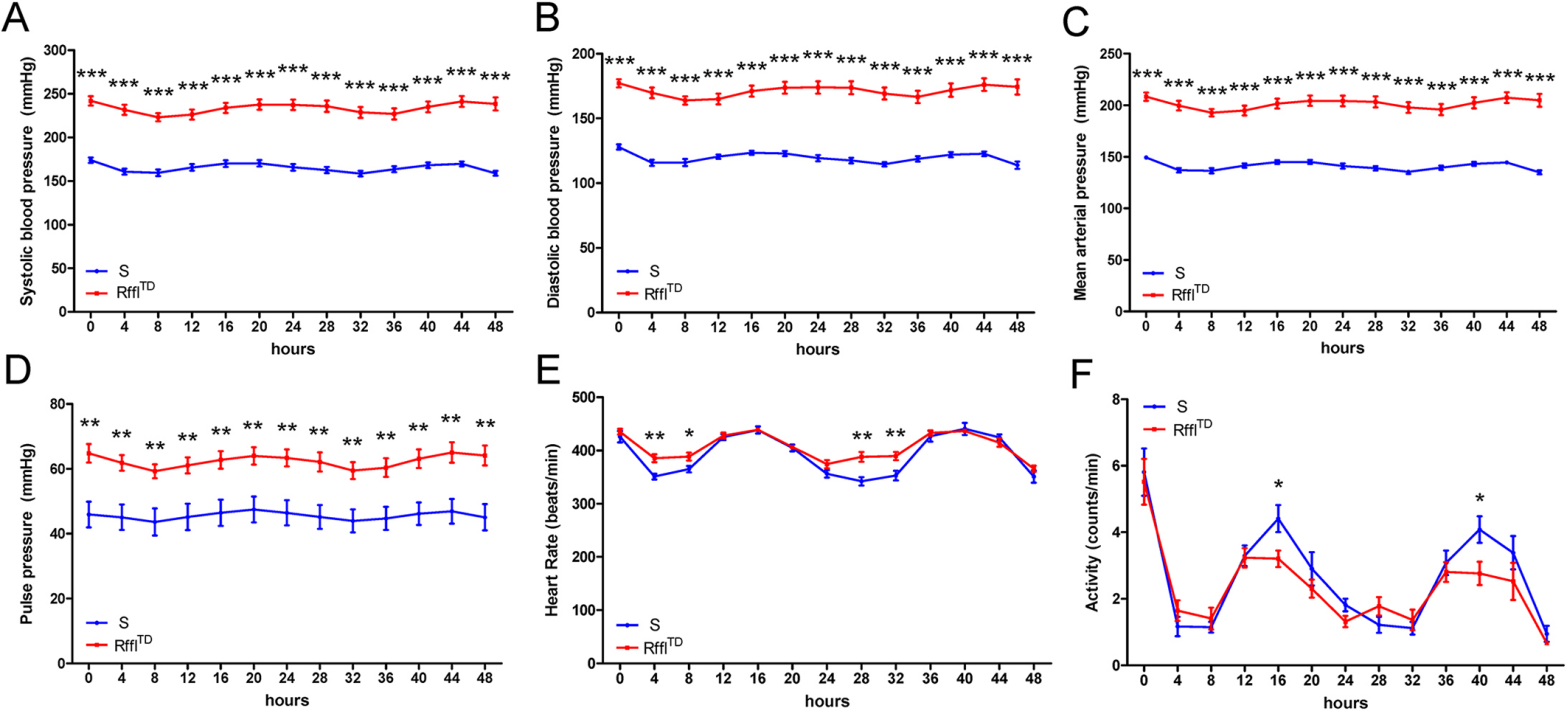
**Higher blood pressure, faster heart rate and less physical activity were observed in Rffl^TD^ compared with S.** (A) Systolic blood pressure. (B) Diastolic blood pressure. (C) Mean arterial pressure. (D) Pulse pressure. (E) Heart rate. (F) Activity. Values are expressed as mean±s.e.m. *P<0.05, **P<0.01, ***P<0.001 (Student's *t*-test). *n*=9 rats (∼14 weeks of age) per group.

**Fig. 6.**
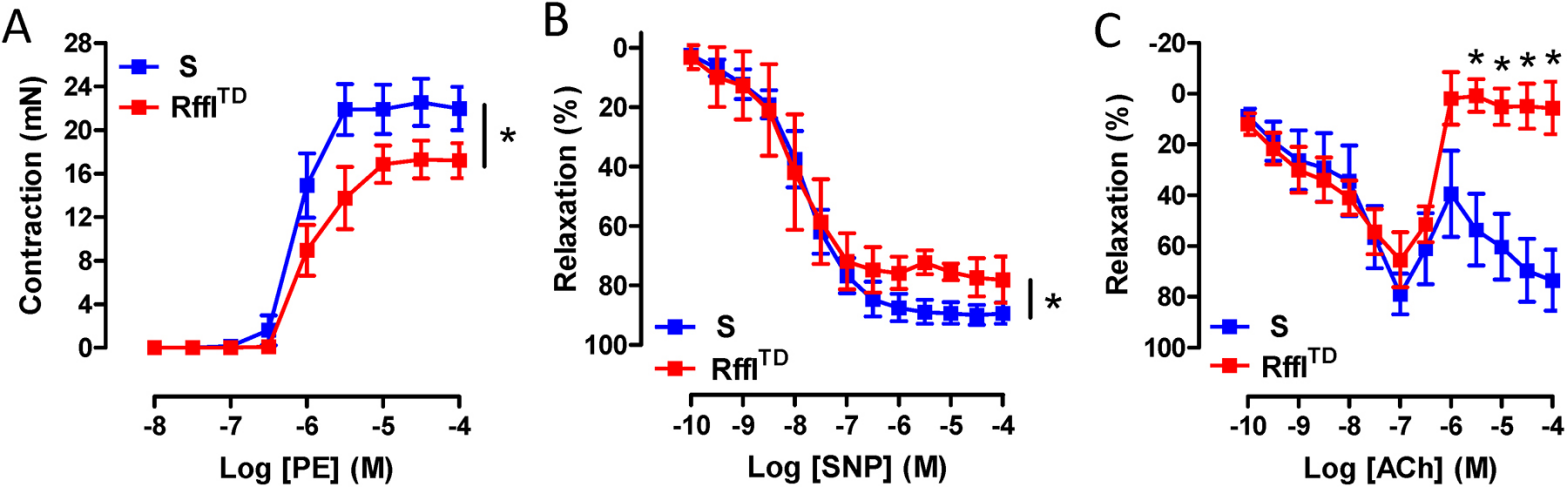
**Resistance arteries from Rffl^TD^ rats presented hypocontractility and impaired endothelium-independent/-dependent relaxation.** (A) Contractile responses to phenylephrine (PE) in mesenteric resistance arteries of S and Rffl^TD^. Non-linear regression analysis (E_max_): *P<0.05. (B) Endothelium-independent relaxation to sodium nitroprusside (SNP) in mesenteric resistance arteries of S and Rffl^TD^. Non-linear regression analysis (E_max_): *P<0.05. (C) Endothelium-dependent relaxation to acetylcholine (ACh) in mesenteric resistance arteries of S and Rffl^TD^. *P<0.05 (two-way ANOVA). Values are expressed as mean±s.e.m. *n*=4-6 rats (∼15 weeks of age) per group.

**Fig. 7.**
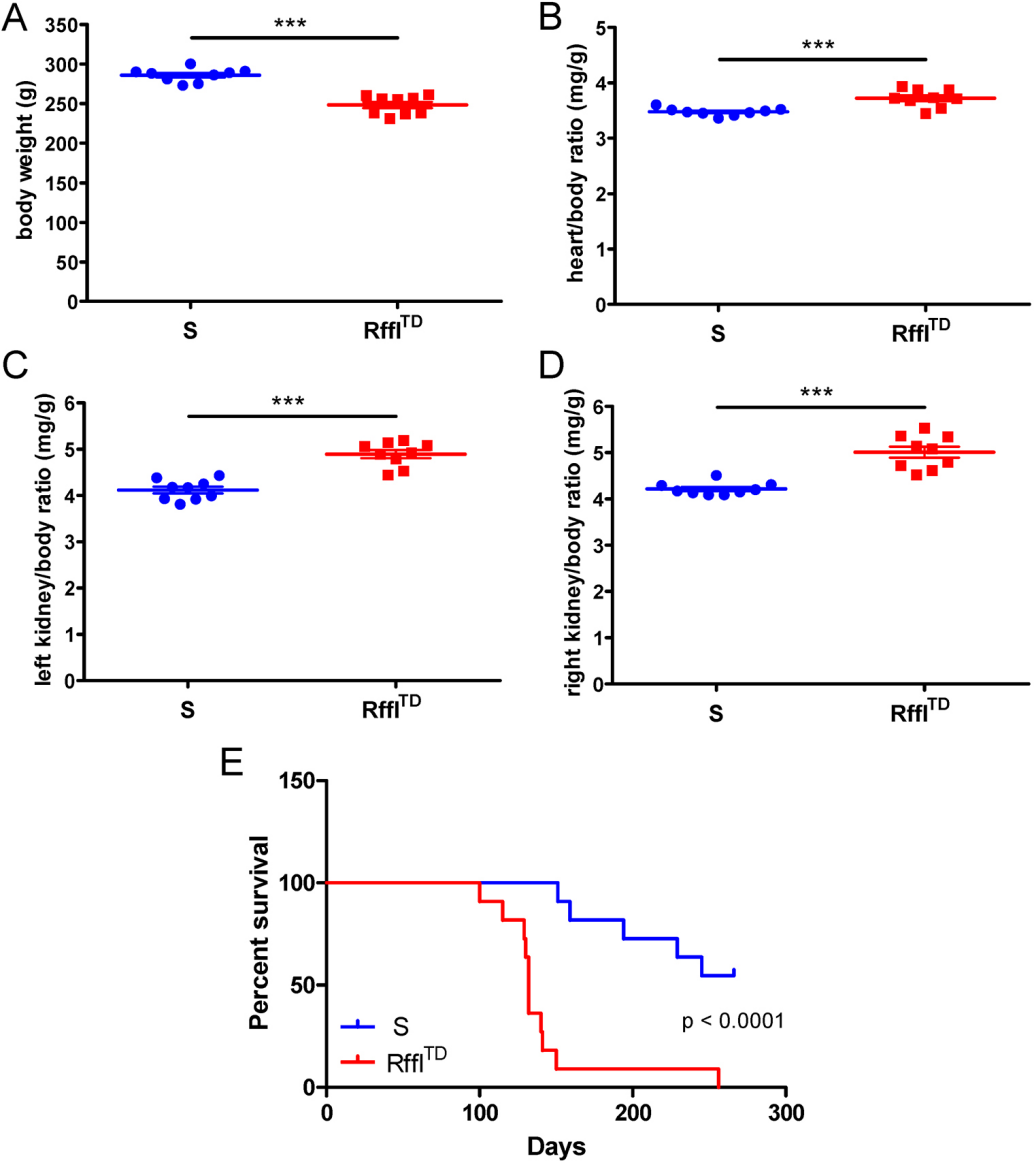
**Lower body weight, worse heart hypertrophy, worse kidney hypertrophy and shorter survival were observed in Rffl^TD^ compared with S.** (A) Body weight. (B) Heart/body weight ratio. (C) Left kidney/body weight ratio. (D) Right kidney/body weight ratio. (E) Kaplan–Meier survival curve. Tissue and body weights (*n*=9 rats per group) were collected at ∼9 weeks of age. The Gehan–Breslow–Wilcoxon test was used to calculate the *P*-value in the survival curve. Values are expressed as mean±s.e.m. ***P<0.001 (Student's *t*-test).

Blood pressure, heart rate and physical activity were further measured by radiotelemetry. Higher blood pressure, faster heartbeat and less physical activity were observed in Rffl^TD^ compared with S at 14 weeks of age (Fig. 5). Vascular reactivity experiments performed on isolated mesenteric resistance arteries indicated significant vascular dysfunction in Rffl^TD^, represented by decreased contractile responses to phenylephrine (PE) (Fig. 6A), impaired endothelium-independent relaxation to sodium nitroprusside (SNP) (Fig. 6B) and impaired endothelium-dependent relaxation to acetylcholine (ACh) (Fig. 6C). When another batch of rats was euthanized at ∼9 weeks of age, Rffl^TD^ already had significantly lower body weight accompanied by severe heart and kidney hypertrophy compared with S (Fig. 7A-D). As we noticed more natural deaths of Rffl^TD^ than S rats during the temporal study, we performed a survival study using the same batch of rats as for the temporal study, and a Kaplan–Meier survival curve showed that Rffl^TD^ had a significantly shorter life span compared with S (Fig. 7E). Therefore, an early increased RDW in Rffl^TD^ preceded a wide range of morbid events in cardiovascular and renal systems.

### The expression of local genes in the 50 bp disrupted region of Rffl^TD^

**Fig. 8.**
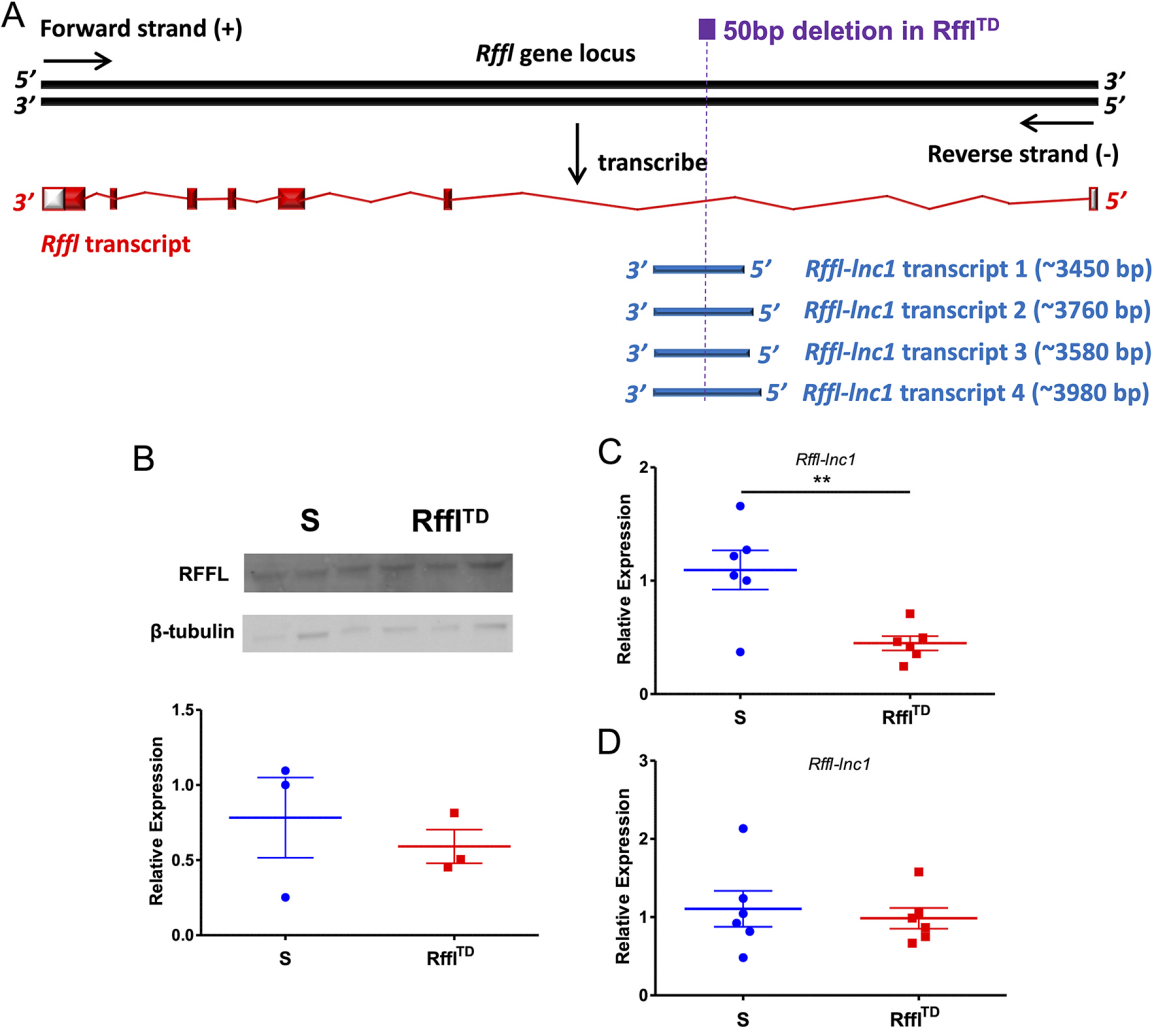
**Expression of RFFL and *Rffl-lnc1* in S and Rffl^TD^.** (A) Schematic showing the protein-coding gene *Rffl* and four isoforms of the long non-coding RNA, *Rffl-lnc1*, in the 50 bp disrupted region (Cheng et al., 2017). The 50 bp region is located at chr10:70188071-70188120 (Genome Assembly: Rnor_6.0). The sequences of four *Rffl-lnc1* isoforms in Dahl salt-sensitive rat (SS/Jr) are provided in the Supplementary Information. (B) Western blot of RFFL using the heart tissue of high-salt-fed rats at ∼10 weeks of age. *n*=3 rats per group. (C) Quantitative real-time PCR of *Rffl-lnc1* using the heart tissue of high-salt-fed rats at ∼10 weeks of age. *n*=6 rats per group. (D) Quantitative real-time PCR of *Rffl-lnc1* using the heart tissue of low-salt-fed rats at ∼9 weeks of age. *n*=6 rats per group. Values are expressed as mean±s.e.m. **P<0.01 (Student's *t*-test).

As a 50 bp deletion is located in the 5′UTR intronic region of the protein-coding gene *Rffl* and the long non-coding RNA, *Rffl-lnc1* (Cheng et al., 2017) (Fig. 8A), the expression of these local genes was further investigated. The proteomics study (Fig. 1), performed on rats fed a high-salt diet, did not identify differential expression of RFFL between S and Rffl^TD^. The western blot study also confirmed that RFFL was not differentially expressed between S and Rffl^TD^ (Fig. 8B). Using the same batch of rats (∼10 weeks of age) fed a high-salt diet, quantitative real-time PCR was performed to measure the expression of *Rffl-lnc1* and the results indicated significantly lower expression of *Rffl-lnc1* in Rffl^TD^ than in S (Fig. 8C). As the current phenotypic studies (Figs 2-7) were performed on low-salt-fed rats, our results suggested that the phenotypic differences in Rffl^TD^ were independent of salt. Therefore, *Rffl-lnc1* expression was further tested using the heart tissue of low-salt-fed rats (∼9 weeks of age), and, interestingly, no significant difference in *Rffl-lnc1* expression was observed between S and Rffl^TD^ (Fig. 8D). Since a significant increase in RDW in Rffl^TD^ was observed as early as 5 weeks of age, the abnormal expression of *Rffl-lnc1* is unlikely to be the cause of the increased RDW. However, other possibilities due to the 50 bp genomic disruption, such as the structural change of *Rffl-lnc1* and interfered interactions between different chromosomal and genomic regions, cannot be ruled out, which will be further discussed below.

## DISCUSSION

RDW measurement is provided as part of the standard test in CBC. Increased RDW has been reported to be associated with a variety of diseases, suggesting its important prognostic value in clinical application. However, investigations of abnormal RDW have been limited to clinical association studies, as increased RDW was usually identified after the patients presented with certain disease symptoms; thus it is difficult to track the temporal causes of increased RDW and physiological disorders in clinical patients. Moreover, genetic causes of increased RDW and its mechanism in disease development are largely unknown. Therefore, a laboratory animal model of an increased RDW trait is urgently needed. We previously reported a CRISPR/Cas9-based genome-engineering rat model, referred to as Rffl^TD^, with a 50 bp deletion within the *Rffl* gene on the genomic background of the Dahl salt-sensitive rat (S) (Cheng et al., 2017). Rffl^TD^ had severe hypertension compared with its control wild-type S rat (Cheng et al., 2017), and the unbiased quantitative proteomics study suggested that Rffl^TD^ also had hematologic disease due to the downregulation of haptoglobin and HGB (Fig. 1), which was further confirmed by the standard CBC test in a separate batch of animals (Fig. 2). Further, a temporal study was performed for a complete evaluation of hematologic, cardiovascular and renal parameters across different ages. Serendipitously, increased RDW was identified in Rffl^TD^ as early as 5 weeks of age (shortly after weaning), followed by other cardiovascular, renal and hematologic syndromes developed at later stages (Figs 3-7). Therefore, our study demonstrates that increased RDW can occur in the early life stage and potentially contribute to eventual cardiovascular and renal comorbidities.

As the primary genomic difference between Rffl^TD^ and its control S rat is the 50 bp within the *Rffl* gene, our study demonstrates that these 50 bp serve as the quantitative trait nucleotides for RDW, which further supported a recent human genome-wide association study (GWAS) showing that increased RDW is inheritable (Pilling et al., 2017). Moreover, the GWAS catalog lists hundreds of human genetic loci associated with RDW, and, interestingly, the single-nucleotide polymorphism rs1046321 is located within 0.5 Mb of the human *RFFL* gene (Buniello et al., 2019). However, limited studies have focused on experimental validation of genetic components of RDW, as there was no reliable genetic and phenotypic animal model of the RDW trait. Thus, our study is the first to experimentally pinpoint a genetic region for increased RDW and to provide a novel genetic rat model with an early genetic onset of increased RDW followed by later cardio-renal comorbidities. Importantly, our study further supports the clinical association of increased RDW with poor cardiovascular and renal outcome in patients (Felker et al., 2007; Förhécz et al., 2009; Pascual-Figal et al., 2009; Lippi et al., 2008; Yonemoto et al., 2018; Wang et al., 2018), indicating that an early genetic predisposition to increased RDW is likely to be the cause of cardio-renal comorbid progression.

The quantitative trait nucleotides of the 50 bp are located in several genes, including a protein-coding gene, *Rffl*, and a long non-coding RNA, *Rffl-lnc1*, with four different isoforms identified on the genomic background of the inbred Dahl salt-sensitive rat (Cheng et al., 2017) (Fig. 8A). Therefore, we examined the expression of these two genes in the heart, but no significant difference in *Rffl* and *Rffl-lnc1* expression was observed at the time of the observation of increased RDW in Rffl^TD^ (Fig. 8B,D), suggesting that the abnormal expression of these two genes is unlikely to cause increased RDW. However, the expression of *Rffl* and *Rffl-lnc1* was only tested in heart tissue; thus, the abnormal expression of these two genes may occur in other tissues. Moreover, we cannot rule out the possibility that the structural modification of *Rffl-lnc1* due to the 50 bp deletion mediates increased RDW in Rffl^TD^. Interestingly, there are other genes, e.g. circular RNAs and *RAD51L3-RFFL* Readthrough, located in the human homologous region of the rat *Rffl* gene. Therefore, further studies are needed to profile these genes in rat and compare their expression between S and Rffl^TD^. The *Rffl* gene is located on rat chromosome 10, and an early report demonstrated an interaction between rat chromosome 2 and rat chromosome 10 (Rapp et al., 1998), suggesting that the genomic disruption in the *Rffl* genomic region may also influence global chromosome conformation. Overall, a short genomic deletion of 50 bp may not only influence the structure and expression of local transcripts, but also cause dysregulated chromosome conformation across the genome. Therefore, our study can only connect the genetic cause of increased RDW with a small genomic region of the 50 bp within the *Rffl* 5′UTR intronic region. As molecular mechanisms of increased RDW are still largely unknown, our Rffl^TD^ model provides an excellent opportunity to understand the pathophysiological roles of RDW in the etiology of cardio-renal diseases.

## MATERIALS AND METHODS

### Animals and diet

All animal procedures and protocols described in this study were approved by the University of Toledo Institutional Animal Care and Use Committee. Animal experiments were performed in accordance with the Guide for the Care and Use of Laboratory Animals. The inbred Dahl salt-sensitive (SS/Jr or S) rat strain and the Rffl^TD^ model were from stocks maintained in our animal facility at our institution. Rats were weaned at 28-30 days of age and fed with a low-salt diet (0.3% NaCl, TD7034, Harlan Teklad). A high-salt diet (2% NaCl, TD94217, Harlan Teklad) was used for experiments involving a high-salt regimen. Only male rats were used, in order to match and extend from the previous study (Cheng et al., 2017) conducted using male rats. In each phenotypic study, any different experimental rat groups were concomitantly bred and co-housed to minimize environmental effects.

### Quantitative mass spectrometry

For protein extraction, at ∼6 weeks of age, the Rffl^TD^ and S rats were switched to a high-salt diet. Heart tissues were collected from the Rffl^TD^ rats (*n*=5, 77 days of age, animal IDs: 47521, 47523, 47524, 47525, 47530) and S rats (*n*=5, 76-77 days of age, animal IDs: S9731, S9734, S9745, S9746, S9750). The tissues were washed with PBS to avoid blood contamination before performing lysis, and 1 ml ice-cold RIPA buffer, containing protease inhibitor cocktail (Thermo Fisher Scientific), was added to 30 mg tissue. The tissue was homogenized and incubated on ice for 10 min. The homogenized tissue was centrifuged at ∼13,000 ***g***, 4°C for 30 min to pellet cell debris and the protein-containing supernatant was transferred to a new clean tube.

Tandem mass tag (TMT) labeling was performed using TMT-10plex isobaric reagents according to the manufacturer's protocol with minor modifications (Thermo Fisher Scientific). Briefly, 95 μg protein from each sample was reduced with dithiothreitol (5 mM) at 45°C for 1 h followed by alkylation with 2-chloroacetamide (15 mM) at room temperature for 30 min. Proteins were precipitated by adding six volumes of ice-cold acetone and incubating overnight at −20°C. Precipitated proteins were pelleted by centrifuging at 8000 ***g*** for 10 min at 4°C and the supernatant was discarded. The pellet was resuspended in 100 μl of 100 mM triethylammonium bicarbonate and digested overnight at 37°C by adding 2 µg sequencing-grade, modified porcine trypsin (Promega, V5113). TMT reagents were reconstituted in 41 µl anhydrous acetonitrile and digested peptides were transferred to the TMT reagent vial and incubated at room temperature for 1 h. The samples of the Rffl^TD^ group were labeled with TMT channels 126, 127N, 128N, 129N and 130N, while the samples of the S group were labeled with TMT channels 127C, 129C, 130C, 128C and 131. The reaction was quenched by adding 8 µl of 5% hydroxylamine and incubating it for a further 15 min. All samples were combined and dried. Prior to mass spectrometry analysis, 100 μg of the peptides was fractionated (ten fractions) using a high-pH reverse-phase fractionation kit following the manufacturer's protocol (Pierce, 84868). Fractions were dried and reconstituted in 12 μl loading buffer (0.1% formic acid and 2% acetonitrile).

For liquid chromatography-mass spectrometry analysis, in order to obtain more accurate quantitation, the multinotch-MS3 method was employed (McAlister et al., 2014). An Orbitrap Fusion (Thermo Fisher Scientific) and RSLC Ultimate 3000 nano-UPLC (Dionex) were used to acquire the data. Two microliters of each fraction were resolved on a nano-capillary reverse-phase column (Acclaim PepMap C18, 2 μm, 75 μm inner diameter × 50 cm, Thermo Fisher Scientific) using a 0.1% formic/acetonitrile gradient at 300 nl/min (2-22% acetonitrile in 150 min; 22-32% acetonitrile in 40 min; 20 min wash at 90% followed by 50 min re-equilibration) and directly sprayed onto the Orbitrap Fusion using an Easy-Spray source (Thermo Fisher Scientific). The mass spectrometer was set to collect one MS1 scan [Orbitrap; 120K resolution; automatic gain control (AGC) target 2×10^5^; maximum ionization time (max IT) 100 ms] followed by data-dependent, ‘Top Speed’ (3 s) MS2 scans [collision-induced dissociation; ion trap; normalized collision energy (NCE) ∼35%; AGC 5×10^3^; max IT 100 ms]. For multinotch-MS3, the top ten precursors from each MS2 were fragmented by high-energy C-trap dissociation (HCD) followed by Orbitrap analysis (NCE ∼55%; 60K resolution; AGC 5×10^4^; max IT 120 ms; 100-500 m/z scan range).

A Proteome Discoverer (v2.1; Thermo Fisher Scientific) was used for data analysis. MS2 spectra were searched against the TrEMBL *Rattus* protein database (released 13 April 2016; 27,785 sequences) using the following search parameters: MS1 and MS2 tolerance were set to 10 ppm and 0.6 Da, respectively; carbamidomethylation of cysteines (57.02146 Da) and TMT labeling of lysine and N-termini of peptides (229.16293 Da) were considered static modifications; oxidation of methionine (15.9949 Da) and deamidation of asparagine and glutamine (0.98401 Da) were considered variable. Identified proteins and peptides were filtered to retain only those that passed ≤1% false-discovery rate threshold. Quantitation was performed using high-quality MS3 spectra (average signal-to-noise ratio of 10, <30% isolation interference, and data were normalized against total peptide).

### Proteomic pathway analysis

Using the DEPs with the Benjamini–Hochberg adjusted *P*-value <0.05 and fold change ≥1.5, IPA (Qiagen) was performed to investigate significantly altered pathways in Rffl^TD^.

### Measurements of hematologic and cardio-renal parameters

Blood was collected through retro-orbital bleeding. A Microtainer^®^ Blood Collection Tube with K2EDTA (Becton Dickinson) was used to store the blood for the immediate measurement of CBC by VETSCAN HM5 (ABAXIS), which includes the measurements of HGB, RBC counts, MCV, MCHC and RDW. A Microtainer^®^ Tube with Serum Separator Additive BD Microgard Closure (Becton Dickinson) was used to store the blood for serum separation. Serum was separated by centrifuging at ∼3000 ***g*** for 10 min at 4°C. BNP level was measured using a Rat BNP 45 ELISA Kit (Abcam) according to the manufacturer's protocol. Serum creatinine level was measured using a Creatinine Assay Kit (Abcam) according to the manufacturer's protocol.

### Blood pressure measurements by radiotelemetry

Blood pressure was recorded and analyzed using radiotelemetry transmitters (HD-S10), receivers and software from Data Sciences International, as described previously (Saad et al., 2007). Briefly, experimental rats were surgically implanted with the transmitters through the femoral artery. Post-surgical rats were individually housed and allowed to recover for 3 days before recording blood pressure. Plotted data were obtained by telemetry recording once every 5 min continuously and averaged for 4-h intervals.

### Myograph

Third-order mesenteric resistance arteries were mounted onto wire myographs (Danish MyoTech, Aarhus, Denmark) in physiological salt solution containing: 130.0 mM NaCl, 4.7 mM KCl, 1.2 mM KH_2_PO_4_, 1.2 mM MgSO_4_·7H_2_O, 14.9 mM NaHCO_3_, 5.5 mM glucose, 1.6 mM CaCl_2_·2H_2_O and 0.03 mM EDTA (all Millipore Sigma). Arteries were normalized to their optimal lumen diameter for active force development, as described previously (McCarthy et al., 2015; Mulvany and Halpern, 1976). Viability of all arteries was initially confirmed by contraction to KCl (120 mM). Endothelium integrity was then tested with a PE-induced contraction (3 μM) followed by endothelium-dependent vasodilation with ACh (3 μM). Cumulative concentration-response curves were performed to α_1_-adrenergic agonist PE (0.1 nM-100 μM), endothelium-independent vasodilator SNP (0.1 nM-100 μM) and ACh (0.1 nM-100 μM) (all Millipore Sigma). Relaxation concentration-response curves were performed after an initial contraction with PE (10 μM). Results are presented as force (mN) for PE and percentage relaxation (%) for ACh and SNP.

### Western blotting

Proteins were isolated from the heart tissues using the CelLytic MT Cell Lysis Reagent and protocol (Sigma-Aldrich). Protein concentrations were calculated using BCA assay. Protein (75 µg) from each sample, as well as 10 µl of ladder (Bio-Rad, Precision Plus Protein Standards) was loaded into the gel. The membrane was blotted for control β-tubulin (Cell Signaling Technology, 2128S; 1:1000) and RFFL (Abcam, ab47994; 1:1000). Chemiluminescence was used for detection. Quantification was performed with ImageJ 1.50i software.

### Quantitative real-time PCR

Total RNA was extracted from the heart tissues using an RNeasy Plus Mini Kit (Qiagen) according to the manufacturer's protocol. cDNA was obtained through reverse transcription with SuperScript III (Invitrogen) using random primers. Quantitative real-time PCR was performed on a QuantStudio™ 5 Real-Time PCR System (Thermo Fisher Scientific) using Power SYBR Green PCR Master Mix (Applied Biosystems). Each experimental group consisted of six biological replicates, each of which had three individual technical replicates. The expression levels of *Rffl-lnc1* (forward primer, 5′-AGCTTGGCTTTTATGGACAAAG-3′; reverse primer, 5′-ACAGCTGAAGGAGACATTAGCAA-3′) relative to the housekeeping gene *Actb* (forward primer, 5′-CCGCGAGTACAACCTTCTTG-3′; reverse primer, 5′-GCAGCGATATCGTCATCCAT-3′) were calculated by the 2^−ΔΔCT^ method.

### Statistical analysis

For proteomic data, unpaired two-tailed Student's *t*-test was performed followed by the Benjamini–Hochberg procedure using R software. Unpaired two-tailed Student's *t*-tests were used for statistical analyses in CBC, serum BNP assay, serum creatinine assay, radiotelemetry measurements, body weight, tissue/body weight ratio, western blot and quantitative real-time PCR. In the vascular reactivity experiments, concentration response curves were analyzed using either non-linear regression analysis (E_max_) or two-way ANOVA. Data were presented as mean±s.e.m. *P*<0.05 was considered to be statistically significant.

## Supplementary Material

Supplementary information

## References

[DMM044081C1] Bilal A., Farooq J. H., Kiani I., Assad S., Ghazanfar H. and Ahmed I. (2016). Importance of mean red cell distribution width in hypertensive patients. *Cureus* 8, e902 10.7759/cureus.902 28070471 PMC5208582

[DMM044081C2] Brandt R. R., Wright R. S., Redfield M. M. and Burnett J. C. Jr (1993). Atrial natriuretic peptide in heart failure. *J. Am. Coll. Cardiol.* 22 Suppl. A, A86-A92. 10.1016/0735-1097(93)90468-G 8376700

[DMM044081C3] Buniello A., MacArthur J. A. L., Cerezo M., Harris L. W., Hayhurst J., Malangone C., McMahon A., Morales J., Mountjoy E., Sollis E. et al. (2019). The NHGRI-EBI GWAS Catalog of published genome-wide association studies, targeted arrays and summary statistics 2019. *Nucleic Acids Res.* 47, D1005-D1012. 10.1093/nar/gky1120 30445434 PMC6323933

[DMM044081C4] Cheng X., Waghulde H., Mell B., Morgan E. E., Pruett-Miller S. M. and Joe B. (2017). Positional cloning of quantitative trait nucleotides for blood pressure and cardiac QT-interval by targeted CRISPR/Cas9 editing of a novel long non-coding RNA. *PLoS Genet.* 13, e1006961 10.1371/journal.pgen.1006961 28827789 PMC5578691

[DMM044081C5] Felker G. M., Allen L. A., Pocock S. J., Shaw L. K., McMurray J. J. V., Pfeffer M. A., Swedberg K., Wang D., Yusuf S., Michelson E. L. et al. (2007). Red cell distribution width as a novel prognostic marker in heart failure: data from the CHARM Program and the Duke Databank. *J. Am. Coll. Cardiol.* 50, 40-47. 10.1016/j.jacc.2007.02.067 17601544

[DMM044081C6] Förhécz Z., Gombos T., Borgulya G., Pozsonyi Z., Prohászka Z. and Jánoskuti L. (2009). Red cell distribution width in heart failure: prediction of clinical events and relationship with markers of ineffective erythropoiesis, inflammation, renal function, and nutritional state. *Am. Heart J.* 158, 659-666. 10.1016/j.ahj.2009.07.024 19781428

[DMM044081C7] Koma Y., Onishi A., Matsuoka H., Oda N., Yokota N., Matsumoto Y., Koyama M., Okada N., Nakashima N., Masuya D. et al. (2013). Increased red blood cell distribution width associates with cancer stage and prognosis in patients with lung cancer. *PLoS ONE* 8, e80240 10.1371/journal.pone.0080240 24244659 PMC3823700

[DMM044081C8] Lippi G., Targher G., Montagnana M., Salvagno G. L., Zoppini G. and Guidi G. C. (2008). Relationship between red blood cell distribution width and kidney function tests in a large cohort of unselected outpatients. *Scand. J. Clin. Lab. Invest.* 68, 745-748. 10.1080/00365510802213550 18618369

[DMM044081C9] Lippi G., Turcato G., Cervellin G. and Sanchis-Gomar F. (2018). Red blood cell distribution width in heart failure: a narrative review. *World J. Cardiol.* 10, 6-14. 10.4330/wjc.v10.i2.6 29487727 PMC5827617

[DMM044081C10] Lu Y.-A., Fan P.-C., Lee C.-C., Wu V. C.-C., Tian Y.-C., Yang C.-W., Chen Y.-C. and Chang C.-H. (2017). Red cell distribution width associated with adverse cardiovascular outcomes in patients with chronic kidney disease. *BMC Nephrol.* 18, 361 10.1186/s12882-017-0766-4 29237417 PMC5729452

[DMM044081C11] McAlister G. C., Nusinow D. P., Jedrychowski M. P., Wühr M., Huttlin E. L., Erickson B. K., Rad R., Haas W. and Gygi S. P. (2014). MultiNotch MS3 enables accurate, sensitive, and multiplexed detection of differential expression across cancer cell line proteomes. *Anal. Chem.* 86, 7150-7158. 10.1021/ac502040v 24927332 PMC4215866

[DMM044081C12] McCarthy C. G., Wenceslau C. F., Goulopoulou S., Ogbi S., Baban B., Sullivan J. C., Matsumoto T. and Webb R. C. (2015). Circulating mitochondrial DNA and Toll-like receptor 9 are associated with vascular dysfunction in spontaneously hypertensive rats. *Cardiovasc. Res.* 107, 119-130. 10.1093/cvr/cvv137 25910936 PMC4560046

[DMM044081C13] Mulvany M. J. and Halpern W. (1976). Mechanical properties of vascular smooth muscle cells in situ. *Nature* 260, 617-619. 10.1038/260617a0 1264225

[DMM044081C14] Pascual-Figal D. A., Bonaque J. C., Redondo B., Caro C., Manzano-Fernandez S., Sánchez-Mas J., Garrido I. P. and Valdes M. (2009). Red blood cell distribution width predicts long-term outcome regardless of anaemia status in acute heart failure patients. *Eur. J. Heart Fail* 11, 840-846. 10.1093/eurjhf/hfp109 19696056

[DMM044081C15] Pilling L. C., Atkins J. L., Duff M. O., Beaumont R. N., Jones S. E., Tyrrell J., Kuo C.-L., Ruth K. S., Tuke M. A., Yaghootkar H. et al. (2017). Red blood cell distribution width: genetic evidence for aging pathways in 116,666 volunteers. *PLoS ONE* 12, e0185083 10.1371/journal.pone.0185083 28957414 PMC5619771

[DMM044081C16] Rapp J. P., Garrett M. R. and Deng A. Y. (1998). Construction of a double congenic strain to prove an epistatic interaction on blood pressure between rat chromosomes 2 and 10. *J. Clin. Invest.* 101, 1591-1595. 10.1172/JCI2251 9541488 PMC508739

[DMM044081C17] Saad Y., Yerga-Woolwine S., Saikumar J., Farms P., Manickavasagam E., Toland E. J. and Joe B. (2007). Congenic interval mapping of RNO10 reveals a complex cluster of closely-linked genetic determinants of blood pressure. *Hypertension* 50, 891-898. 10.1161/HYPERTENSIONAHA.107.097105 17893371

[DMM044081C18] Sanchez-Chaparro M. A., Calvo-Bonacho E., Gonzalez-Quintela A., Cabrera M., Sainz J. C., Fernandez-Labandera C., Aguado L. Q., Meseguer A. F., Valdivielso P., Roman-Garcia J. et al. (2010). Higher red blood cell distribution width is associated with the metabolic syndrome: results of the Ibermutuamur CArdiovascular RIsk assessment study. *Diabetes Care* 33, e40 10.2337/dc09-1707 20190288

[DMM044081C19] Shao Q., Korantzopoulos P., Letsas K. P., Tse G., Hong J., Li G. and Liu T. (2018). Red blood cell distribution width as a predictor of atrial fibrillation. *J. Clin. Lab. Anal.* 32, e22378 10.1002/jcla.22378 29315856 PMC6817116

[DMM044081C20] Wang B., Lu H., Gong Y., Ying B. and Cheng B. (2018). The association between red blood cell distribution width and mortality in critically ill patients with acute kidney injury. *Biomed. Res. Int.* 2018, 9658216 10.1155/2018/9658216 30345313 PMC6174796

[DMM044081C21] Yonemoto S., Hamano T., Fujii N., Shimada K., Yamaguchi S., Matsumoto A., Kubota K., Hashimoto N., Oka T., Senda M. et al. (2018). Red cell distribution width and renal outcome in patients with non-dialysis-dependent chronic kidney disease. *PLoS ONE* 13, e0198825 10.1371/journal.pone.0198825 29889895 PMC5995355

