# Supplementary material for: Genetic predisposition for increased red blood cell distribution width is an early risk factor for cardiovascular and renal comorbidities
Source: Dis Model Mech. 2020 May 18;13(5):dmm044081. doi: 10.1242/dmm.044081 (PMC7325433; doi:10.1242/dmm.044081)
Supplement: Supplementary information [file dmm-13-044081-s1.pdf]

Rffl-lnc1 Isoform – 1

GAGGUUCUAGGUGUUUGGGUACUAGAUCGAGGCGUGUAGUAGUCCCACUAUGGGAUAUGGGAUGUUAGAGGUUUUUUUUUUCUU
UUUAACCAAAGAAACCAUUCAGGAAGCCACAGGCCUUCCACCAACUGCUAAUUUUGAAUCCCUGAGGGCUUUUCUACAGCUGAAGGA
GACAUUAGCAAUUAGUGGAGUCCCGCCUUCCUAUUUUCCAGAUGAGGAGACAGACCUGUAAGAAACAUAAAUGGCUUUGUCCAUAAA
AGCCAAGCUAUUGUGCCCAGUGUUUACUCCUGACACUGAAUGUCGCCUGGGGAACUUCCAGCACUUGGAAAGCCAUGGAGUUAGGCC
AUAGGCCAGACCUGCGUGAGGGCGUCAGGCCUGUCACAUGGAUGAGGCCGCUGCAGUGCUCAACAGGAGCUGCUGGGGUGGUGGCA
GGUUUGGUUCAGGGCAGUGUGUACCACAGGACCCUUUGAAUAUUCUCAGACACUUUUCUGGAGAACUGUUCUUUGGGGACCAGCUG
UCAGAAAUCGUCUGCAUUUGAGUGGGGGGCCAAGGUUUAAUUAUAAAACAUUUCCAACUUCUGCGGCUCUUUUUUUUUUUUUUUU
CCCAGCCUUCAUACUCCACGUUUUGUUUUGGACUUCCUUUCCUAAUUUCAGCACCAAUUGGUACAUUUUUAUUGCUCCAUUGCAGG
GCAGAUGGUAGCACAAGGCAUGGGAAAUCCAGAAAAUGUCUUAGAAGUCCGUGACUGUUAUGAGUGCUUGCCAGGCAGAUCUUCAA
CUUUAAAUAGUAAUAGAUUUUCCCGUCCAGUGAGCCAGUUGUCAGAAACUAUGAAAAGUCACCUGUUUGUCCAAGGUCUAAUUCUG
AGCUGGAGAGCUGUCAGACAACCUUGGACAGGAAGUUGGGUUCAAGCAGAAUGUGCUUGUCUUCACUGCAGCCUGGGUGUCUGCAG
UUUGAAACUUGCUGCGGGUAAAAAGUCAAGACUUCAACCAGAUGUGGUGGUGCACACCUGUAAUUAUUUCAGCAUCCAGGAGGCGG
AGGGAGGAAUAUCUUGGAGGCCAAGACUUUUAAUAUCGAAAUGUUGUCCCUGUGUGAGAGGGAGGUAAUACAGUAGCCAUUCAAUA
CACAGGAGUUUCUUUUCCAGUGCUGUGACCCGUGCCUGACACACAGCAGUUUAGGGGAGAAAAACUUUUGGUUUUCUUCUUGUCUU
UUGGCUUACAGUUUGUGGAUACGCUGUCUGCCUUGACAGGAAGUGCUUGGUUCGAGGAACGUACCUAGUCGUAUUGCAUCUGCAG
UAAUAAGGAAGCAAAGAGUAAGACCAGUAGGGCUUGGCUAUGACUUCAAGGCAAGCCCAGAGCGCUGACUUCCCCUUGUGAGGCUCC
AUCUUCUUUAAGUUUCACAGCCUUCCAAAACAGCCAUCAUCUGGGGACACUAGAGCCUAUGGGGACAUUUCAUGUUCAACCACAAAA
CAGCUGUUGCCUCUCUCUGUACAUAGCAGCUUUUCUUCUGAGACCCAGGUCAUUCUUUGGAGUCACAGGCAUGGUGUUUUCCCUGA
GUUCUACAAUGUUGCAGUUUAUAAACCGAAAGGGCACUGAGCGGACUGCUGCUGGCCUGCUACUUUUGGUAGGUGUGAGAUUCAGU
CUGUGCUAGCUCUUUUUUAAAAUAACAAUAGGGAAUUUUAGCAUAGAAUUCACCAGACCUUUGAGUAACAGGUUCCUUUUGUGAGG
CUCCAGGAUUCCUAGCACCUUCUCAGUGUGUUUUGACCAAGGUAGUUUACAAAGUACUGUGAGUCUUUGAGAAGGUUUUUCAUGU
UUGAAUUAAGAGUUUCCUUGCAUACUGGAGGUUGGAUCUAAUGCUGGUCCCUCAUCUGCCCCCCCACCCCACCCCCCAGUCUCUGGA
GAAGCUAUUAAAUAAGUAAUUUGCCUUGGGGUCACUCAGCUGGUAAACAAUCUCGACAGCCCCUUUUUCCUAAGCCCUACUCCCUUU
UUCAUUGCUUCCUGCUGUGAAAUUGCUUCAUUGCCUUCAUUAGGGGACCAGAUUGGCUGUGGGAUAGCCACCUGCAGGGAGUCACC
UUGGAAUCUUUGUGGAUUAUUAAAACUGCUUGGGUUAUUUCAAAACUGGUCCAUUCUGCUGUCCUGGGCUAUAAUUAGGAGCAGA
UAAAGGUUUUCUUUUAGAUUAUCAUUGUCUAAGAAAUACAUUUUUAUUGUCCUUGUCCUAAUUCACUGGGAUUGUGGGCUACUAA
AGCAGGCACCUCCUCGAGCAAGUCCCAUGAACACCCCUGAAACCAUCAUUUCCUUUUGUUUCAGCGUAGAAUUGUCUGGGAAUACUU
AGGUAUCGGUGACAGCCUAGGUCACUGAACUACCCGUGCCCUUCUUUGUGCACAGAGUACGCUUGGUUCAGUAUAUUGAACUGAGC
AUGCCCAGAGCACCUGUGCUUCCUAGGUAAAAGUCUUGAUUACAUUCUUUUUUCUUGCAUUCAGUAUCCCAGCCAAAGAAGUCUAGG
GGGUGGGAGGUGAGUGUUGGGUCGGUGCUGGGCUGGCUUUGCAAAUCAGAGAGGACAGUACUUCCUGAAAUUAGCACCAGGGUCG
AUGUUAUCAAGGAUGUGAAUAGGAACUGUUUUGUAUCUUGUCGUGGGAUAGUGACAAAGUAAUAUGUGAGGGCCAGUUUAAGCAC
AGGCGUUCUUUGGUAGGGGAGAAUAUGGUAUAAUCACAGAGGAGGGAGGGUGGUUGUUGGUGUUGGUUUAUUUUUUGUGGUAC
UAGCAGACUAGAAUUUUUCUUUUGCUUUUUGGGCUGGUUUCUGGGCAUGCCACUUCUGGCAGGAGCAGAAAUUUCUGCAGAAAGU
CAGCAUAAUUUUUAGAUUUGAUAAAACAUCAGAGCAAGCUAAAAGUUGGGAGGAAGAAAGUGCUUGGUCCGAAUUUCUCCUUUAUC
ACUGACUUGCAUCCAGCUGCGGUAUGGUGCUGCUGUCACACCCCCCACCCCCACCCCCACCCCCAGGCCAGCCGAUGAGAAUGCUCAUU
CUACCCCGACCUGGUAGAGAAGCACAGAGAGGAAGACCAGCGGAAACUUACCGCAAUGGAGUAGAAAGGGGAAGUAAUGUCACAGCC
UAGCCUGCGGGAGGACAACCAUGUGGGCAGCUCCGGGAAGAUCUUCAAGGGGUUCCCACACUCAUUUAGAGCCAGUGGAAAAGGCCA
CUAAGAUCAUUGAGCCUUUUGGUAAGAACUAGAUUGAACUGGAUUAAAGGUUUUGGCUUGUCUAAUGGAGGUAACAACAACAACCC
AAAACAAAGACGGGAUCUUUGUCUUCUAAAAACCUUCAAAAGCCAGGUGUGUAUCUGGUGAGGUGGGAGGGUUGCUUGAGUCCGGG
AGUUCAAGAUUAGCCUCAGAAACAUAGUUUCCAGCUGGAAAACAGAAGUAAAAACCUUUAAAAACAAACAAAAAAAAAAAAAAAAA
AAAAAAAAA

Disease Models & Mechanisms • Supplementary information

Rffl-lnc1 Isoform – 2

GUCCCUUUCCUUUCCGAGAUGCUUGCCGUGAUCUCCUCUUGCACUGACUCACAUCUUGUCUUCGUUCCCUUUUCUCACCCAGAUUUU
GCCUGAUUCUUUUCUCCCAUUAACAGUCACUGUGUGGGUGUCCUGCCUGCUACUCACCUCCGCCAUAUCCCAACCCAUCCACGAGUCA
UAUAAUUAGCAUGGGAGCCAAGUGUGAUCACAUUAAAAACCUAAUAGAAAGACCGUAUAUGGAGAACAGUCCUUUAGGUUCAUCAG
UGGUUUGCUUUCAAGCAGGAUAUUUUCUGACCGGAGUUGAAAGGGAAGAGGAGGUUCUAGGUGUUUGGGUACUAGAUCGAGGCGU
GUAGUAGUCCCACUAUGGGAUAUGGGAUGUUAGAGGUUUUUUUUUUCUUUUUAACCAAAGAAACCAUUCAGGAAGCCACAGGCCUU
CCACCAACUGCUAAUUUUGAAUCCCUGAGGGCUUUUCUACAGCUGAAGGAGACAUUAGCAAUUAGUGGAGUCCCGCCUUCCUAUUUU
CCAGAUGAGGAGACAGACCUGUAAGAAACAUAAAUGGCUUUGUCCAUAAAAGCCAAGCUAUUGUGCCCAGUGUUUACUCCUGACACU
GAAUGUCGCCUGGGGAACUUCCAGCACUUGGAAAGCCAUGGAGUUAGGCCAUAGGCCAGACCUGCGUGAGGGCGUCAGGCCUGUCAC
AUGGAUGAGGCCGCUGCAGUGCUCAACAGGAGCUGCUGGGGUGGUGGCAGGUUUGGUUCAGGGCAGUGUGUACCACAGGACCCUUU
GAAUAUUCUCAGACACUUUUCUGGAGAACUGUUCUUUGGGGACCAGCUGUCAGAAAUCGUCUGCAUUUGAGUGGGGGGCCAAGGUU
UAAUUAUAAAACAUUUCCAACUUCUGCGGCUCUUUUUUUUUUUUUUUUCCCAGCCUUCAUACUCCACGUUUUGUUUUGGACUUCCU
UUCCUAAUUUCAGCACCAAUUGGUACAUUUUUAUUGCUCCAUUGCAGGGCAGAUGGUAGCACAAGGCAUGGGAAAUCCAGAAAAUG
UCUUAGAAGUCCGUGACUGUUAUGAGUGCUUGCCAGGCAGAUCUUCAACUUUAAAUAGUAAUAGAUUUUCCCGUCCAGUGAGCCAG
UUGUCAGAAACUAUGAAAAGUCACCUGUUUGUCCAAGGUCUAAUUCUGAGCUGGAGAGCUGUCAGACAACCUUGGACAGGAAGUUG
GGUUCAAGCAGAAUGUGCUUGUCUUCACUGCAGCCUGGGUGUCUGCAGUUUGAAACUUGCUGCGGGUAAAAAGUCAAGACUUCAAC
CAGAUGUGGUGGUGCACACCUGUAAUUAUUUCAGCAUCCAGGAGGCGGAGGGAGGAAUAUCUUGGAGGCCAAGACUUUUAAUAUCG
AAAUGUUGUCCCUGUGUGAGAGGGAGGUAAUACAGUAGCCAUUCAAUACACAGGAGUUUCUUUUCCAGUGCUGUGACCCGUGCCUG
ACACACAGCAGUUUAGGGGAGAAAAACUUUUGGUUUUCUUCUUGUCUUUUGGCUUACAGUUUGUGGAUACGCUGUCUGCCUUGAC
AGGAAGUGCUUGGUUCGAGGAACGUACCUAGUCGUAUUGCAUCUGCAGUAAUAAGGAAGCAAAGAGUAAGACCAGUAGGGCUUGGC
UAUGACUUCAAGGCAAGCCCAGAGCGCUGACUUCCCCUUGUGAGGCUCCAUCUUCUUUAAGUUUCACAGCCUUCCAAAACAGCCAUCA
UCUGGGGACACUAGAGCCUAUGGGGACAUUUCAUGUUCAACCACAAAACAGCUGUUGCCUCUCUCUGUACAUAGCAGCUUUUCUUCU
GAGACCCAGGUCAUUCUUUGGAGUCACAGGCAUGGUGUUUUCCCUGAGUUCUACAAUGUUGCAGUUUAUAAACCGAAAGGGCACUG
AGCGGACUGCUGCUGGCCUGCUACUUUUGGUAGGUGUGAGAUUCAGUCUGUGCUAGCUCUUUUUUAAAAUAACAAUAGGGAAUUU
UAGCAUAGAAUUCACCAGACCUUUGAGUAACAGGUUCCUUUUGUGAGGCUCCAGGAUUCCUAGCACCUUCUCAGUGUGUUUUGACC
AAGGUAGUUUACAAAGUACUGUGAGUCUUUGAGAAGGUUUUUCAUGUUUGAAUUAAGAGUUUCCUUGCAUACUGGAGGUUGGAUC
UAAUGCUGGUCCCUCAUCUGCCCCCCCACCCCACCCCCCAGUCUCUGGAGAAGCUAUUAAAUAAGUAAUUUGCCUUGGGGUCACUCA
GCUGGUAAACAAUCUCGACAGCCCCUUUUUCCUAAGCCCUACUCCCUUUUUCAUUGCUUCCUGCUGUGAAAUUGCUUCAUUGCCUUC
AUUAGGGGACCAGAUUGGCUGUGGGAUAGCCACCUGCAGGGAGUCACCUUGGAAUCUUUGUGGAUUAUUAAAACUGCUUGGGUUA
UUUCAAAACUGGUCCAUUCUGCUGUCCUGGGCUAUAAUUAGGAGCAGAUAAAGGUUUUCUUUUAGAUUAUCAUUGUCUAAGAAAU
ACAUUUUUAUUGUCCUUGUCCUAAUUCACUGGGAUUGUGGGCUACUAAAGCAGGCACCUCCUCGAGCAAGUCCCAUGAACACCCCUG
AAACCAUCAUUUCCUUUUGUUUCAGCGUAGAAUUGUCUGGGAAUACUUAGGUAUCGGUGACAGCCUAGGUCACUGAACUACCCGUG
CCCUUCUUUGUGCACAGAGUACGCUUGGUUCAGUAUAUUGAACUGAGCAUGCCCAGAGCACCUGUGCUUCCUAGGUAAAAGUCUUG
AUUACAUUCUUUUUUCUUGCAUUCAGUAUCCCAGCCAAAGAAGUCUAGGGGGUGGGAGGUGAGUGUUGGGUCGGUGCUGGGCUGG
CUUUGCAAAUCAGAGAGGACAGUACUUCCUGAAAUUAGCACCAGGGUCGAUGUUAUCAAGGAUGUGAAUAGGAACUGUUUUGUAUC
UUGUCGUGGGAUAGUGACAAAGUAAUAUGUGAGGGCCAGUUUAAGCACAGGCGUUCUUUGGUAGGGGAGAAUAUGGUAUAAUCAC
AGAGGAGGGAGGGUGGUUGUUGGUGUUGGUUUAUUUUUUGUGGUACUAGCAGACUAGAAUUUUUCUUUUGCUUUUUGGGCUGG
UUUCUGGGCAUGCCACUUCUGGCAGGAGCAGAAAUUUCUGCAGAAAGUCAGCAUAAUUUUUAGAUUUGAUAAAACAUCAGAGCAAG
CUAAAAGUUGGGAGGAAGAAAGUGCUUGGUCCGAAUUUCUCCUUUAUCACUGACUUGCAUCCAGCUGCGGUAUGGUGCUGCUGUCA
CACCCCCCACCCCCACCCCCACCCCCAGGCCAGCCGAUGAGAAUGCUCAUUCUACCCCGACCUGGUAGAGAAGCACAGAGAGGAAGACCA
GCGGAAACUUACCGCAAUGGAGUAGAAAGGGGAAGUAAUGUCACAGCCUAGCCUGCGGGAGGACAACCAUGUGGGCAGCUCCGGGAA
GAUCUUCAAGGGGUUCCCACACUCAUUUAGAGCCAGUGGAAAAGGCCACUAAGAUCAUUGAGCCUUUUGGUAAGAACUAGAUUGAAC
UGGAUUAAAGGUUUUGGCUUGUCUAAUGGAGGUAACAACAACAACCCAAAACAAAGACGGGAUCUUUGUCUUCUAAAAACCUUCAAA
AGCCAGGUGUGUAUCUGGUGAGGUGGGAGGGUUGCUUGAGUCCGGGAGUUCAAGAUUAGCCUCAGAAACAUAGUUUCCAGCUGGA
AAACAGAAGUAAAAACCUUUAAAAACAAACAAAAAAAAAAAAAAAAAAAAAAAAAAA

Disease Models & Mechanisms • Supplementary information

Rffl-lnc1 Isoform – 3

AUAAUUAGCAUGGGAGCCAAGUGUGAUCACAUUAAAAACCUAAUAGAAAGACCGUAUAUGGAGAACAGUCCUUUAGGUUCAUCAGU
GGUUUGCUUUCAAGCAGGAUAUUUUCUGACCGGAGUUGAAAGGGAAGAGGAGGUUCUAGGUGUUUGGGUACUAGAUCGAGGCGUG
UAGUAGUCCCACUAUGGGAUAUGGGAUGUUAGAGGUUUUUUUUUCUUUUUAACCAAAGAAACCAUUCAGGAAGCCACAGGCCUUC
CACCAACUGCUAAUUUUGAAUCCCUGAGGGCUUUUCUACAGCUGAAGGAGACAUUAGCAAUUAGUGGAGUCCCGCCUUCCUAUUUUC
CAGAUGAGGAGACAGACCUGUAAGAAACAUAAAUGGCUUUGUCCAUAAAAGCCAAGCUAUUGUGCCCAGUGUUUACUCCUGACACUG
AAUGUCGCCUGGGGAACUUCCAGCACUUGGAAAGCCAUGGAGUUAGGCCAUAGGCCAGACCUGCGUGAGGGCGUCAGGCCUGUCACA
UGGAUGAGGCCGCUGCAGUGCUCAACAGGAGCUGCUGGGGUGGUGGCAGGUUUGGUUCAGGGCAGUGUGUACCACAGGACCCUUUG
AAUAUUCUCAGACACUUUUCUGGAGAACUGUUCUUUGGGGACCAGCUGUCAGAAAUCGUCUGCAUUUGAGUGGGGGGCCAAGGUUU
AAUUAUAAAACAUUUCCAACUUCUGCGGCUCUUUUUUUUUUUUUUUUCCCAGCCUUCAUACUCCACGUUUUGUUUUGGACUUCCUU
UCCUAAUUUCAGCACCAAUUGGUACAUUUUUAUUGCUCCAUUGCAGGGCAGAUGGUAGCACAAGGCAUGGGAAAUCCAGAAAAUGU
CUUAGAAGUCCGUGACUGUUAUGAGUGCUUGCCAGGCAGAUCUUCAACUUUAAAUAGUAAUAGAUUUUCCCGUCCAGUGAGCCAGU
UGUCAGAAACUAUGAAAAGUCACCUGUUUGUCCAAGGUCUAAUUCUGAGCUGGAGAGCUGUCAGACAACCUUGGACAGGAAGUUGG
GUUCAAGCAGAAUGUGCUUGUCUUCACUGCAGCCUGGGUGUCUGCAGUUUGAAACUUGCUGCGGGUAAAAAGUCAAGACUUCAACC
AGAUGUGGUGGUGCACACCUGUAAUUAUUUCAGCAUCCAGGAGGCGGAGGGAGGAAUAUCUUGGAGGCCAAGACUUUUAAUAUCGA
AAUGUUGUCCCUGUGUGAGAGGGAGGUAAUACAGUAGCCAUUCAAUACACAGGAGUUUCUUUUCCAGUGCUGUGACCCGUGCCUGA
CACACAGCAGUUUAGGGGAGAAAAACUUUUGGUUUUCUUCUUGUCUUUUGGCUUACAGUUUGUGGAUACGCUGUCUGCCUUGACA
GGAAGUGCUUGGUUCGAGGAACGUACCUAGUCGUAUUGCAUCUGCAGUAAUAAGGAAGCAAAGAGUAAGACCAGUAGGGCUUGGCU
AUGACUUCAAGGCAAGCCCAGAGCGCUGACUUCCCCUUGUGAGGCUCCAUCUUCUUUAAGUUUCACAGCCUUCCAAAACAGCCAUCAU
CUGGGGACACUAGAGCCUAUGGGGACAUUUCAUGUUCAACCACAAAACAGCUGUUGCCUCUCUCUGUACAUAGCAGCUUUUCUUCUG
AGACCCAGGUCAUUCUUUGGAGUCACAGGCAUGGUGUUUUCCCUGAGUUCUACAAUGUUGCAGUUUAUAAACCGAAAGGGCACUGA
GCGGACUGCUGCUGGCCUGCUACUUUUGGUAGGUGUGAGAUUCAGUCUGUGCUAGCUCUUUUUUAAAAUAACAAUAGGGAAUUUU
AGCAUAGAAUUCACCAGACCUUUGAGUAACAGGUUCCUUUUGUGAGGCUCCAGGAUUCCUAGCACCUUCUCAGUGUGUUUUGACCA
AGGUAGUUUACAAAGUACUGUGAGUCUUUGAGAAGGUUUUUCAUGUUUGAAUUAAGAGUUUCCUUGCAUACUGGAGGUUGGAUC
UAAUGCUGGUCCCUCAUCUGCCCCCCCACCCCACCCCCCAGUCUCUGGAGAAGCUAUUAAAUAAGUAAUUUGCCUUGGGGUCACUCA
GCUGGUAAACAAUCUCGACAGCCCCUUUUUCCUAAGCCCUACUCCCUUUUUCAUUGCUUCCUGCUGUGAAAUUGCUUCAUUGCCUUC
AUUAGGGGACCAGAUUGGCUGUGGGAUAGCCACCUGCAGGGAGUCACCUUGGAAUCUUUGUGGAUUAUUAAAACUGCUUGGGUUA
UUUCAAAACUGGUCCAUUCUGCUGUCCUGGGCUAUAAUUAGGAGCAGAUAAAGGUUUUCUUUUAGAUUAUCAUUGUCUAAGAAAU
ACAUUUUUAUUGUCCUUGUCCUAAUUCACUGGGAUUGUGGGCUACUAAAGCAGGCACCUCCUCGAGCAAGUCCCAUGAACACCCCUG
AAACCAUCAUUUCCUUUUGUUUCAGCGUAGAAUUGUCUGGGAAUACUUAGGUAUCGGUGACAGCCUAGGUCACUGAACUACCCGUG
CCCUUCUUUGUGCACAGAGUACGCUUGGUUCAGUAUAUUGAACUGAGCAUGCCCAGAGCACCUGUGCUUCCUAGGUAAAAGUCUUG
AUUACAUUCUUUUUUCUUGCAUUCAGUAUCCCAGCCAAAGAAGUCUAGGGGGUGGGAGGUGAGUGUUGGGUCGGUGCUGGGCUGG
CUUUGCAAAUCAGAGAGGACAGUACUUCCUGAAAUUAGCACCAGGGUCGAUGUUAUCAAGGAUGUGAAUAGGAACUGUUUUGUAUC
UUGUCGUGGGAUAGUGACAAAGUAAUAUGUGAGGGCCAGUUUAAGCACAGGCGUUCUUUGGUAGGGGAGAAUAUGGUAUAAUCAC
AGAGGAGGGAGGGUGGUUGUUGGUGUUGGUUUAUUUUUUGUGGUACUAGCAGACUAGAAUUUUUCUUUUGCUUUUUGGGCUGG
UUUCUGGGCAUGCCACUUCUGGCAGGAGCAGAAAUUUCUGCAGAAAGUCAGCAUAAUUUUUAGAUUUGAUAAAACAUCAGAGCAAG
CUAAAAGUUGGGAGGAAGAAAGUGCUUGGUCCGAAUUUCUCCUUUAUCACUGACUUGCAUCCAGCUGCGGUAUGGUGCUGCUGUCA
CACCCCCCACCCCCACCCCCACCCCCAGGCCAGCCGAUGAGAAUGCUCAUUCUACCCCGACCUGGUAGAGAAGCACAGAGAGGAAGACCA
GCGGAAACUUACCGCAAUGGAGUAGAAAGGGGAAGUAAUGUCACAGCCUAGCCUGCGGGAGGACAACCAUGUGGGCAGCUCCGGGAA
GAUCUUCAAGGGGUUCCCACACUCAUUUAGAGCCAGUGGAAAAGGCCACUAAGAUCAUUGAGCCUUUUGGUAAGAACUAGAUUGAAC
UGGAUUAAAGGUUUUGGCUUGUCUAAUGGAGGUAACAACAACAACCCAAAACAAAGACGGGAUCUUUGUCUUCUAAAAACCUUCAAA
AGCCAGGUGUGUAUCUGGUGAGGUGGGAGGGUUGCUUGAGUCCGGGAGUUCAAGAUUAGCCUCAGAAACAUAGUUUCCAGCUGGA
AAACAGAAGUAAAAACCUUUAAAAACAAACAAAAAAAAAAAAAAAAAAAAAAAAAAAA

Disease Models & Mechanisms • Supplementary information

Rffl-lnc1 Isoform – 4

GGCCUGUCACUGGGAGUUCACAUUGUCCCCUGAGUCUAACAGCAGAGCUUCAUUUGUGGCACCUUUAUGUCACCGUGCAUUAUCUGC
CUUUUCUUUUAGGAGCUGUGGCCUCGCCUUUAUCCCACUGAGCGGAUUUAUCCCCUGGUGUCCAGUUCUGUUCCCUGCACAGUAGG
CCUUCAGUGCCAGUCACAUGAAUAGGUGGAGCUUUGACUGACAUUUGUCCCUUUCCUUUCCGAGAUGCUUGCCGUGAUCUCCUCUU
GCACUGACUCACAUCUUGUCUUCGUUCCCUUUUCUCACCCAGAUUUUGCCUGAUUCUUUUCUCCCAUUAACAGUCACUGUGUGGGU
GUCCUGCCUGCUACUCACCUCCGCCAUAUCCCAACCCAUCCACGAGUCAUAUAAUUAGCAUGGGAGCCAAGUGUGAUCACAUUAAAAA
CCUAAUAGAAAGACCGUAUAUGGAGAACAGUCCUUUAGGUUCAUCAGUGGUUUGCUUUCAAGCAGGAUAUUUUCUGACCGGAGUUG
AAAGGGAAGAGGAGGUUCUAGGUGUUUGGGUACUAGAUCGAGGCGUGUAGUAGUCCCACUAUGGGAUAUGGGAUGUUAGAGGUU
UUUUUUUUCUUUUUAACCAAAGAAACCAUUCAGGAAGCCACAGGCCUUCCACCAACUGCUAAUUUUGAAUCCCUGAGGGCUUUUCUA
CAGCUGAAGGAGACAUUAGCAAUUAGUGGAGUCCCGCCUUCCUAUUUUCCAGAUGAGGAGACAGACCUGUAAGAAACAUAAAUGGCU
UUGUCCAUAAAAGCCAAGCUAUUGUGCCCAGUGUUUACUCCUGACACUGAAUGUCGCCUGGGGAACUUCCAGCACUUGGAAAGCCAU
GGAGUUAGGCCAUAGGCCAGACCUGCGUGAGGGCGUCAGGCCUGUCACAUGGAUGAGGCCGCUGCAGUGCUCAACAGGAGCUGCUG
GGGUGGUGGCAGGUUUGGUUCAGGGCAGUGUGUACCACAGGACCCUUUGAAUAUUCUCAGACACUUUUCUGGAGAACUGUUCUUU
GGGGACCAGCUGUCAGAAAUCGUCUGCAUUUGAGUGGGGGGCCAAGGUUUAAUUAUAAAACAUUUCCAACUUCUGCGGCUCUUUUU
UUUUUUUUUUUCCCAGCCUUCAUACUCCACGUUUUGUUUUGGACUUCCUUUCCUAAUUUCAGCACCAAUUGGUACAUUUUUAUUGC
UCCAUUGCAGGGCAGAUGGUAGCACAAGGCAUGGGAAAUCCAGAAAAUGUCUUAGAAGUCCGUGACUGUUAUGAGUGCUUGCCAGG
CAGAUCUUCAACUUUAAAUAGUAAUAGAUUUUCCCGUCCAGUGAGCCAGUUGUCAGAAACUAUGAAAAGUCACCUGUUUGUCCAAGG
UCUAAUUCUGAGCUGGAGAGCUGUCAGACAACCUUGGACAGGAAGUUGGGUUCAAGCAGAAUGUGCUUGUCUUCACUGCAGCCUGG
GUGUCUGCAGUUUGAAACUUGCUGCGGGUAAAAAGUCAAGACUUCAACCAGAUGUGGUGGUGCACACCUGUAAUUAUUUCAGCAUC
CAGGAGGCGGAGGGAGGAAUAUCUUGGAGGCCAAGACUUUUAAUAUCGAAAUGUUGUCCCUGUGUGAGAGGGAGGUAAUACAGUA
GCCAUUCAAUACACAGGAGUUUCUUUUCCAGUGCUGUGACCCGUGCCUGACACACAGCAGUUUAGGGGAGAAAAACUUUUGGUUUU
CUUCUUGUCUUUUGGCUUACAGUUUGUGGAUACGCUGUCUGCCUUGACAGGAAGUGCUUGGUUCGAGGAACGUACCUAGUCGUAU
UGCAUCUGCAGUAAUAAGGAAGCAAAGAGUAAGACCAGUAGGGCUUGGCUAUGACUUCAAGGCAAGCCCAGAGCGCUGACUUCCCCU
UGUGAGGCUCCAUCUUCUUUAAGUUUCACAGCCUUCCAAAACAGCCAUCAUCUGGGGACACUAGAGCCUAUGGGGACAUUUCAUGUU
CAACCACAAAACAGCUGUUGCCUCUCUCUGUACAUAGCAGCUUUUCUUCUGAGACCCAGGUCAUUCUUUGGAGUCACAGGCAUGGUG
UUUUCCCUGAGUUCUACAAUGUUGCAGUUUAUAAACCGAAAGGGCACUGAGCGGACUGCUGCUGGCCUGCUACUUUUGGUAGGUGU
GAGAUUCAGUCUGUGCUAGCUCUUUUUUAAAAUAACAAUAGGGAAUUUUAGCAUAGAAUUCACCAGACCUUUGAGUAACAGGUUCC
UUUUGUGAGGCUCCAGGAUUCCUAGCACCUUCUCAGUGUGUUUUGACCAAGGUAGUUUACAAAGUACUGUGAGUCUUUGAGAAGG
UUUUUCAUGUUUGAAUUAAGAGUUUCCUUGCAUACUGGAGGUUGGAUCUAAUGCUGGUCCCUCAUCUGCCCCCCCACCCCACCCCC
CAGUCUCUGGAGAAGCUAUUAAAUAAGUAAUUUGCCUUGGGGUCACUCAGCUGGUAAACAAUCUCGACAGCCCCUUUUUCCUAAGCC
CUACUCCCUUUUUCAUUGCUUCCUGCUGUGAAAUUGCUUCAUUGCCUUCAUUAGGGGACCAGAUUGGCUGUGGGAUAGCCACCUGC
AGGGAGUCACCUUGGAAUCUUUGUGGAUUAUUAAAACUGCUUGGGUUAUUUCAAAACUGGUCCAUUCUGCUGUCCUGGGCUAUAA
UUAGGAGCAGAUAAAGGUUUUCUUUUAGAUUAUCAUUGUCUAAGAAAUACAUUUUUAUUGUCCUUGUCCUAAUUCACUGGGAUUG
UGGGCUACUAAAGCAGGCACCUCCUCGAGCAAGUCCCAUGAACACCCCUGAAACCAUCAUUUCCUUUUGUUUCAGCGUAGAAUUGUC
UGGGAAUACUUAGGUAUCGGUGACAGCCUAGGUCACUGAACUACCCGUGCCCUUCUUUGUGCACAGAGUACGCUUGGUUCAGUAUA
UUGAACUGAGCAUGCCCAGAGCACCUGUGCUUCCUAGGUAAAAGUCUUGAUUACAUUCUUUUUUCUUGCAUUCAGUAUCCCAGCCAA
AGAAGUCUAGGGGGUGGGAGGUGAGUGUUGGGUCGGUGCUGGGCUGGCUUUGCAAAUCAGAGAGGACAGUACUUCCUGAAAUUAG
CACCAGGGUCGAUGUUAUCAAGGAUGUGAAUAGGAACUGUUUUGUAUCUUGUCGUGGGAUAGUGACAAAGUAAUAUGUGAGGGCC
AGUUUAAGCACAGGCGUUCUUUGGUAGGGGAGAAUAUGGUAUAAUCACAGAGGAGGGAGGGUGGUUGUUGGUGUUGGUUUAUUU
UUUGUGGUACUAGCAGACUAGAAUUUUUCUUUUGCUUUUUGGGCUGGUUUCUGGGCAUGCCACUUCUGGCAGGAGCAGAAAUUUC
UGCAGAAAGUCAGCAUAAUUUUUAGAUUUGAUAAAACAUCAGAGCAAGCUAAAAGUUGGGAGGAAGAAAGUGCUUGGUCCGAAUUU
CUCCUUUAUCACUGACUUGCAUCCAGCUGCGGUAUGGUGCUGCUGUCACACCCCCCACCCCCACCCCCACCCCCAGGCCAGCCGAUGAG
AAUGCUCAUUCUACCCCGACCUGGUAGAGAAGCACAGAGAGGAAGACCAGCGGAAACUUACCGCAAUGGAGUAGAAAGGGGAAGUAA
UGUCACAGCCUAGCCUGCGGGAGGACAACCAUGUGGGCAGCUCCGGGAAGAUCUUCAAGGGGUUCCCACACUCAUUUAGAGCCAGUG
GAAAAGGCCACUAAGAUCAUUGAGCCUUUUGGUAAGAACUAGAUUGAACUGGAUUAAAGGUUUUGGCUUGUCUAAUGGAGGUAACA
ACAACAACCCAAAACAAAGACGGGAUCUUUGUCUUCUAAAAACCUUCAAAAGCCAGGUGUGUAUCUGGUGAGGUGGGAGGUUGCU
UGAGUCCGGGAGUUCAAGAUUAGCCUCAGAAACAUAGUUUCCAGCUGGAAAACAGAAGUAAAAACCUUUAAAAACAAACAAAAAAAA
AAAAAAAAAAAAAAAAAAAA

Disease Models & Mechanisms • Supplementary information

Table S1

Click here to Download Table S1
